# Changes in Stress Reduction Following a 28-Day Prostate Cancer Patient Empowerment Program (PC-PEP) among Prostate Cancer Survivors

**DOI:** 10.3390/curroncol30090577

**Published:** 2023-08-29

**Authors:** Laura Burge, Gabriela Ilie, Cody MacDonald, Hayley Riel, Rob David Harold Rutledge

**Affiliations:** 1 Undergraduate Medical Education, Faculty of Medicine, Dalhousie University, Halifax, NS B3H 4R2, Canada; lburge@dal.ca; 2 Department of Community Health and Epidemiology, Faculty of Medicine, Dalhousie University, Halifax, NS B3H 1V7, Canada; codymacdonald@dal.ca; 3 Department of Urology, Faculty of Medicine, Dalhousie University, Halifax, NS B3H 2Y9, Canada; 4 Department of Radiation Oncology, Faculty of Medicine, Dalhousie University, Halifax, NS B3H 1V7, Canada; rob.rutledge@nshealth.ca; 5 College of Pharmacy, University of Manitoba, Winnipeg, MB R3E 0T5, Canada; hayley.riel@umanitoba.ca

**Keywords:** prostate cancer, stress reduction, heart rate variability, brain wave activity, PC-PEP, prostate cancer survivorship

## Abstract

Prostate cancer (PCa) survivors often experience post-treatment challenges that impact their well-being and mental health. The Prostate Cancer Patient Empowerment Program (PC-PEP) aims to address these issues through a comprehensive intervention, involving daily meditation/breathing exercises, physical activity, pelvic floor exercises, emotional connection strategies, and peer support. This study presents a secondary analysis of a Phase 2 feasibility study that evaluated the effects of a 28-day PC-PEP intervention on stress reduction. Thirty patients with PCa from the Maritimes, Canada, underwent pre- and post-intervention assessments to measure brainwave activity (delta, theta, alpha, beta, and gamma) using the Muse™ headband, and heart rate variability (HRV) using the HeartMath^®^ Inner Balance™ as indicators of stress reduction. A statistically significant Time × Sensor Scalp Assessment Time interaction emerged for all brain waves. Amplitudes were generally higher during the first half of the meditation assessment time but became comparable afterward. A statistically significant Time × Sensor Scalp Location × Sensor Scalp Assessment Time interaction also emerged for alpha waves, indicating higher prefrontal lobe amplitudes than temporal lobe amplitudes from pre- to post-assessment. There were no statistically significant differences in HRV metrics from pre- to post-intervention, except for a marginally significant achievement score, indicating increased HRV coherence post-intervention. The findings suggest that the stress reduction component of PC-PEP successfully improved outcomes related to decreased stress. These results have implications for the development of future iterations of PC-PEP interventions, aiming to optimize participant benefits.

## 1. Introduction

Prostate cancer (PCa) stands as the most diagnosed cancer among Canadian males, constituting one-fifth of all male cancer diagnoses and affecting 1 in 9 men during their lifetime [1]. Fortunately, given early screening efforts, advancements in technology, and the localized nature of the cancer, the 5-year survival rate is one of the highest of all malignancies, at 91% [1,2]. With markedly high survival rates, PCa survivors represent a vast demographic group in Canada. As such, gaining comprehensive knowledge of the long-term survivorship needs of this population is important.

Despite the promising PCa prognosis, many patients report challenges after undergoing treatment, which has an impact on their quality of life [2,3]. In a study conducted by Palmer, 69% of PCa survivors reported problems with urinary and sexual function after having had their prostate surgically removed [4]. These challenges are common sources of stress for PCa survivors and impact their ability to interact with their friends and family [5]. Additionally, this population has been shown to have higher rates of depression, anxiety, and overall distress symptoms compared to those without a lifetime cancer diagnosis, which are common manifestations of stress [6,7,8]. For example, Chhatre and colleagues surveilled 743 patients with PCa over the course of 24 months and found that rates of depression increased significantly from baseline for all cancer-risk groups (14.9% to 43.5% for low-risk group, 24.9% to 43.2% for intermediate-risk, and 21.6% to 47.1% for high-risk) [9]. Similarly, in another study, 10.9% of PCa patients reported anxiety via the Memorial Anxiety Scale for Prostate Cancer, and 14.1% scored within the anxious range on the Hospital Anxiety and Depression Scale [10]. Moreover, stress can contribute to physical symptoms as well, including disrupted sleep, changes in appetite, fatigue, and difficulties in concentration [11]. These symptoms can have a negative impact on overall well-being and quality of life for men undergoing prostate cancer treatment [12]. It is important to note that addressing stress and providing psychosocial support as part of comprehensive cancer care is crucial. Increased prevalence of urinary/sexual dysfunction, poor mental health, and perceived stress have been shown to worsen the quality of life, which emphasizes the importance of making stress reduction measures a priority for PCa survivors [8,12,13].

Integrating strategies such as meditation, exercise, and social connection can help mitigate the impact of stress on individuals with prostate cancer and improve their overall mental and emotional well-being. Mindfulness meditation, for instance, is widely recognized for its ability to target stress reduction by focusing on thoughts and feelings [14]. For example, participants who followed an 8-week meditation program reported reduced psychological symptoms, heightened sense of control, and increased spiritual experience [15]. Similarly, Carlson et al. implemented a mindfulness-based stress reduction program among breast cancer and PCa patients, resulting in significantly reduced stress symptoms, improved sleep quality, and overall quality of life post-intervention [16].

Exercise is another potent stress reduction tool supported by various studies [17,18,19]. Exercise enhances the body’s stress-coping mechanisms by impacting neurotransmitters, such as dopamine and serotonin, which positively affect mood and behavior [17,18,19]. Breus and O’Connor attribute exercise’s stress reduction to the “time-out” hypothesis, offering a break from stressors and a calming effect [20]. Among patients with a PCa diagnosis, exercise improves their quality of life and lowers all-cause mortality risk [21,22].

Strong support networks are pivotal for well-being and can serve as protective barriers against stress [23]. Social support plays a crucial role in coping with significant stressors like PCa diagnosis and treatment aftermath [24]. Roesch et al. found that relying on social supports improved coping compared to avoidance strategies [25]. Maintaining intimacy in romantic relationships offers an effective approach to stress reduction related to sexuality in PCa survivors [26,27,28].

Implementing an intervention featuring similar strategies holds promise for enhancing the well-being of PCa survivors. Specifically designed to address the most common unmet needs among PCa survivors, the 28-day multifaceted PCa Patient Empowerment Program (PC-PEP) offers a comprehensive approach. This intervention covers various life aspects: stress reduction through daily meditation and breathing exercises, improved physical health via dietary adjustments and prescribed aerobic and strength exercises, enhanced urinary and sexual function through pelvic floor exercises, and boosted social connection via strategies targeting emotional needs [29].

Analysis of primary outcomes from a Phase 2 feasibility study and a Phase 3 randomized clinical trial demonstrated statistically significant improvements in mental and physical health for participants completing the PC-PEP intervention (in 28-day and 6-month formats, respectively) [29,30]. Notably, program compliance analysis underscored participants’ strong motivation to engage fully in all program components [29].

This paper presents a secondary analysis of a Phase 2 feasibility study aimed at evaluating the efficacy of the 28-day PC-PEP intervention in reducing stress. This assessment was conducted by analyzing changes in brain wave activity and heart rate variability (HRV) before and after the intervention, involving a sample of 30 patients with a history of PCa from the Maritimes region of Canada. The study’s primary objective was to explore the feasibility and safety of the 28-day intervention, with mental health as the main focus. Alongside this, secondary outcomes encompass stress reduction, quality of life, urologic function, and physical fitness. While stress reduction outcomes are reported here, other secondary outcomes (physical fitness, program evaluation, and weekly compliance on all aspects of the program) have been addressed in a separate publication [29], while urologic and quality of life outcomes are the subject of another manuscript in preparation. We hypothesized that physiological stress would decrease from pre- to post-28-day PC-PEP intervention. 

## 2. Materials and Methods

The PC-PEP was a 28-day program beginning on 11 January 2019 in Halifax, Nova Scotia, Canada. The sample of participants comprised 30 men with a history of non-metastatic prostate cancer, recruited through Prostate Cancer Support Groups and poster advertisements in the Urology and Radiation Oncology Clinics at the Queen Elizabeth II Health Sciences Centre, Halifax. Inclusion criteria were non-metastatic PCa diagnosis, ability to understand, speak, and write in English, an email address, a cell phone that could receive text messages, and the ability to travel to Dalhousie University in Halifax on three separate occasions. The participants were screened by a physician who determined whether they were eligible to enroll and safe to participate in the program. Characteristics of the sample, methodology, and procedures have been published elsewhere [29].

Prior to their first visit to Dalhousie University (pre-intervention), participants completed an online survey assessing their mental health and physical/functional measures of their quality of life. At their first study visit (between 1 and 3 days pre-intervention), participants underwent baseline assessments, which included strength and fitness measurements conducted by an exercise physiologist and stress levels assessed by a research assistant via a portable EEG system (Muse™) and HRV measurement tool (HeartMath^®^). 

At the second visit, on 11 January 2019, participants attended a half-day seminar that described and demonstrated the elements of the PC-PEP program. The next day, they were started on the daily materials of the program for the following 28 days. The participants were asked to complete weekly compliance surveys on all aspects of the program. Lastly, on 10 February 2019, participants completed all surveys, as well as strength/fitness and stress assessments that were conducted at baseline. All participants had normal or corrected-to-normal vision, no known neurological impairments, and provided written informed consent approved by the Nova Scotia Health Research Ethics Board. The study followed research ethical standards as prescribed in the 1964 Declaration of Helsinki. No adverse events occurred during this study. 

### 2.1. PC-PEP Intervention

The PC-PEP intervention has been described elsewhere [29]. Briefly, the PC-PEP program consisted of daily emails, including video messages from the program’s leads (G.I. and R.R.), which outlined the components of the program for that day and provided dietary and connection recommendations. The physical fitness portion of the PC-PEP program involved adherence to any form of aerobic exercise (biking, dancing, swimming, etc.) for 30 min, 5 times per week, and a combination of bodyweight and resistance band progressive (4 levels of difficulty) interval strength training exercises for 30 min per day, twice per week (Tuesdays and Thursdays). Three resistance bands of varying tension were provided to all participants. The combination of aerobic and strength exercises equated to 150 min or more of moderate to strenuous activity per week, in line with World Health Organization recommendations for healthy adults [31,32]. The pelvic floor muscle training (PMFT) component of PC-PEP consisted of 8-min guided PMFT videos, 3 times per day, which progressively increased in difficulty throughout the program’s duration. The intimacy and connection component was delivered through the daily video messages (via email) and encouraged participants to explore the different ways of meeting emotional needs (e.g., intellectual, emotional, recreational, physical, and self-forms of intimacy), such as calling an old friend and sharing from the heart. The peer accountability aspect of the program involved a buddy system in which two buddies (also participants in the study) were assigned to each participant. Participants were encouraged to call their buddies at least once per week to check on their adherence to the intervention and to discuss any other aspects of the program. Lastly, the stress-reduction component of PC-PEP was comprised of daily mindfulness meditation and deep breathing sessions lasting 10 min. These were guided sessions, using a 10-min pre-recorded instruction video led by the program’s leads (G.I. and R.R.). Weekly compliance on the various aspects of the program has been collected, analyzed, and reported elsewhere [29].

### 2.2. Measures

#### 2.2.1. Brain Wave Activity

Pre- and post-electrophysiological (EEG) recordings of stress reduction via meditation were recorded using the Muse™ EEG headband [33]. Brainwave amplitudes for delta, theta, alpha, beta, and gamma were measured in decibels (dB) with sensors on four locations of the scalp: two frontal (AF7, AF8) and two temporal (TP9, TP10). Data was recorded continuously in the Mind Monitor app for 7 min, with averages generated at 30 s intervals [34].

Several studies demonstrated that Muse™ EEG headbands were effective in measuring event-related brain potential and had strong internal reliability compared to traditional EEG systems [35,36,37]. For example, Ratti and colleagues [36] compared two medical-grade EEG systems with two consumer EEG systems (including Muse™) and found that all four were effective measures of brain wave activity, providing the basis for their usefulness as an inexpensive brain function measurement tool in clinical trials. 

Delta Waves (1–4 Hz): Delta waves have the highest amplitude and lowest frequency of all brainwaves and typically characterize deep (dreamless) sleep and body repair. Higher delta wave amplitudes signify increased non-REM sleep activation, lower arousal, and decreased consciousness. Contrastingly, lower amplitudes signify less relaxation [38,39,40].

Theta Waves (4–8 Hz): Theta waves correspond with creativity, dreams, deep meditation, and reduced consciousness. Theta waves are typically increased during mundane activities such as highway driving, shaving one’s legs, and meditating. Higher theta waves signify lowered focus and increased daydreaming, which often leads to the generation of novel ideas. Reduced theta amplitudes indicate less daydreaming and less idea formation [39,40].

Alpha Waves (8–12 Hz): Alpha waves indicate physical and mental relaxation, as well as creative and artistic ideation. Higher alpha wave amplitudes signify conscious relaxation and reflection, while lower amplitudes represent less relaxation and passive thinking [39,40].

Beta Waves (12–20 Hz): Beta waves are representative of alert, active thinking, such as while participating in conversation. Higher beta wave amplitudes signify arousal and complex thought formation, while lower amplitudes indicate less arousal and decreased active thinking [39,40].

Gamma Waves (30–50 Hz): Gamma waves have the highest frequency and lowest amplitude and are linked to heightened perception, learning, and problem-solving ability. High gamma wave amplitudes could indicate higher stress and arousal, while lower amplitudes may correspond with depression and learning disabilities [40].

#### 2.2.2. Muse™ Metrics 

Muse™ metrics (calm, neutral, birds, and recoveries) were automatically generated from the Muse™ EEG headband data obtained during the 5-min guided meditation. A calm state indicates restful focus on breathing, a neutral state is the natural resting state between poor attention and deep focus, bird sounds were played during extended periods of deep focus on breath, and recoveries were recorded each time attention was brought back to the focus on breathing after the mind had wandered. Points (pt) were awarded for every second spent in a calm or neutral state. Neutral states earned 1 pt/second, while calm states earned 3 pts/second. Birds were recorded as the total number of bird sounds played during the recording, while recoveries were the total number of times participants returned their focus after wandering [41]. Data was exported directly to Statistical Package for the Social Sciences (SPSS; Version 28; SPSS; IBM Corp., Armonk, NY, USA) for analysis. 

#### 2.2.3. Heart Rate Variability 

HRV was measured during a 10-min breathing exercise, using a breath pacer (5 s inhale, 5 s exhale) in the HeartMath^®^ app, “Inner Balance”. Participants attached the Inner Balance™ infrared pulse sensor to their earlobe, which measured their heart rate 125 times per second throughout the exercise and recorded real-time data into the “Inner Balance” app on the research team’s tablet via Bluetooth. An overall HRV coherence score, which was updated every 5s with more stable/regular heart rhythms yielding higher scores, was captured. Scores ranging from 0 to 0.5 indicated low coherence, 0.5 to 0.9 indicated medium coherence, and greater than 0.9 indicated high coherence at the 1st challenge level (for beginners). Low coherence indicates greater irregularity in HRV, which is often associated with stress, anger, and frustration. High coherence indicates greater consistency in HRV, which is often linked to positive emotions, such as love and appreciation [42]. The % of coherence indicates the percentage of time spent in each coherence category (low, medium, high) throughout the session, and the average coherence score was generated by summing all coherence scores and dividing by the number of scores. The achievement score is the total of all coherence scores, measured every 5 s, accumulated throughout the session. An achievement score of 300 is considered a good starting score [43]. Compliance with this aspect of PC-PEP was high (95%) among participants, as described elsewhere [29].

### 2.3. Statistical Analysis

Using the Muse™ Mind Monitor application, data was transmitted via Bluetooth from the headband to a tablet. A CSV file was created and then imported into SPSS. A three-way repeated measures ANOVA with Time (pre-intervention, post-intervention) × Sensor Scalp Locations (TP9, TP10, AF7, AF8) × Sensor Scalp Assessment Time (14 measurements, 30 s apart) was conducted to compare delta, theta, alpha, beta, and gamma waves during the Muse™ recording. A repeated measures ANOVA assessed pre- versus post-stress reduction Muse™ metrics (calm and neutral state points, number of recoveries, and birds heard) and heart rate variability (HeartMath^®^ data). The assumptions of the tests were evaluated and were found tenable except for Mauchley’s test of sphericity, which was violated, *p* < 0.001. Therefore, the Greenhouse–Geiser adjustment for the degrees of freedom was used in data reporting. No missing data was observed.

## 3. Results

### 3.1. Demographic Measures

The demographic characteristics of the sample are reported elsewhere [29] and have been included as a Supplementary Materials. The majority of participants were white (93%), highly educated (university completed, 67%), retired (70%), with an annual household income greater than CAD 80,000 (73%), and a mean age of 68.93 years (range 56–83 years old). All men were in a relationship, and most were 2 years post-PCa diagnosis (73%). Most participants (60%) were treated with radical prostatectomy +/− radiation and hormones. 

### 3.2. Brain Wave Activity

**Figure 1:**
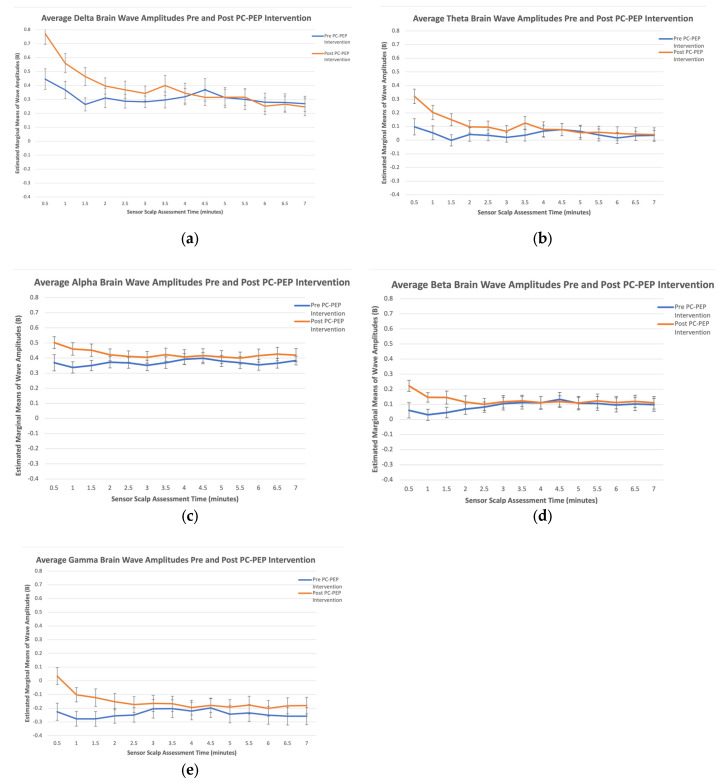
Estimated marginal means of brain wave amplitudes at pre- and post-28-day PC-PEP intervention in a sample of 30 PCa survivors from the Maritimes, Canada. (**a**) Delta brain wave amplitudes across sensor scalp assessment time; (**b**) theta brain wave amplitudes across sensor scalp assessment time; (**c**) alpha brain wave amplitudes across sensor scalp assessment time; (**d**) beta brain wave amplitudes across sensor scalp assessment time; (**e**) gamma brain wave amplitudes across sensor scalp assessment time.

Delta. A statistically significant main effect of Sensor Scalp Location, F (2.11, 57.05) = 19.28, *p* < 0.001, and Sensor Scalp Assessment Time, F (4.38, 118.21) = 11.20, *p* < 0.001 was observed. Most importantly, there was a statistically significant Time × Sensor Scalp Assessment Time interaction, F (4.67, 126.03) = 4.06, *p* = 0.002 (Table 1). Post-delta amplitudes appeared greater (indicating more non-rapid eye-movement activation or low arousal) than pre-amplitudes across the 7 min of meditation for the first half (up to minute 3.5) of the assessment time, but comparable afterward (see Figure 1a). 

Theta. There was a statistically significant main effect of Sensor Scalp Location, F (1.97, 53.18) = 81.79, *p* < 0.001, and Sensor Scalp Assessment Time, F (4.27, 115.15) = 6.74, *p* < 0.001. The results also displayed a statistically significant Time × Sensor Scalp Assessment Time interaction, F (4.67, 126.15) = 4.14, *p* = 0.002. Post-theta amplitudes appeared greater (indicating reduced conscious focus and increased daydreaming) than pre-amplitudes in the first 4 min of meditation but were comparable afterward (see Figure 1b).

**Figure 2:**
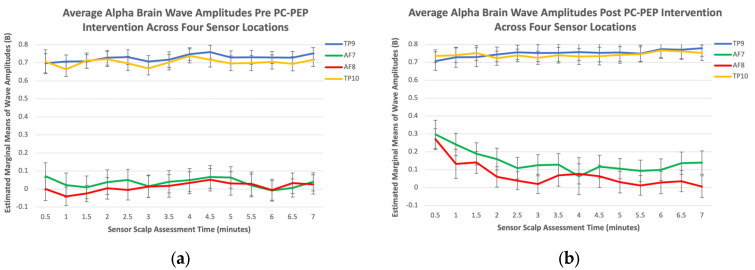
(**a**) Comparison of estimated marginal means of alpha waves across four sensor scalp locations at pre-28-day PC-PEP intervention in a sample of 30 PCa survivors from the Maritimes, Canada; (**b**) comparison of estimated marginal means of alpha waves across four sensor scalp locations at post-28-day PC-PEP intervention in a sample of 30 PCa survivors from the Maritimes, Canada.

Alpha. The results presented a statistically significant main effect of Sensor Scalp Location, F (2.07, 55.87) = 225.15, *p* < 0.001, and Time × Sensor Scalp Assessment Time interaction, F (4.69, 126.60) = 2.44, *p* = 0.04. Post-alpha amplitudes appeared greater (indicating greater physical and mental relaxation, and reflection) than pre-amplitudes in the first 4 min of meditation and comparable to pre-intervention for the remaining duration (see Figure 1c). Interestingly, the results also displayed a significant Time × Sensor Scalp Location × Sensor Scalp Assessment Time interaction, F (7.62, 205.73) = 2.09, *p* = 0.04. Post-alpha amplitudes were greater than pre-amplitudes at the two frontal sensor scalp locations (AF7 and AF8), indicating increased alpha wave activity in the prefrontal cortex across all assessment times, compared to the two temporal sensor scalp locations (TP9 and TP10), which did not demonstrate increased activity across assessment times (see Figure 2). 

Beta. There was a statistically significant main effect of Sensor Scalp Location, F (1.86, 50.11) = 39.72, *p* < 0.001, and Time × Sensor Scalp Assessment Time interaction, F (4.83, 130.32) = 4.48, *p* < 0.001. Post-beta amplitudes appeared greater (indicating increased arousal and complex thought formation) than pre-beta amplitudes up to minute 2.5 and were comparable afterward (see Figure 1d). 

Gamma. A significant main effect of Sensor Scalp Location, F (1.95, 52.65) = 4.49, *p* = 0.02, emerged. There was also a significant Time × Sensor Scalp Assessment Time interaction, F (3.43, 92.72) = 3.62, *p* = 0.01. Post-gamma amplitudes appeared higher (indicating greater stress but lower indications of depression) than pre-amplitudes in the first 3.5 min of meditation (see Figure 1e). Interestingly, there was also a significant Sensor Scalp Location × Sensor Scalp Assessment Time interaction, F (5.30, 143) = 2.37, *p* = 0.04. 

### 3.3. Muse™ Metrics

Repeated measures ANOVA assessing Muse™ metrics (calm, neutral, birds, and recoveries) revealed no statistically significant differences from pre- to post-PC-PEP intervention, *p* > 0.05 (Table 2). 

### 3.4. Heart Rate Variability

Repeated measures ANOVA assessing HRV metrics revealed no statistically significant improvements from pre- to post-PC-PEP intervention, *p* > 0.05. We noted a marginally significant effect for achievement score, *p* = 0.07, revealing that, on average, participants had higher post-PC-PEP HRV coherence when compared to baseline assessments (Table 3).

## 4. Discussion

Previous results have shown that exposure to a short (28 days) or long (6 months) version of PC-PEP results in decreased mental distress and need for clinical treatment [29,30]. Here, we assessed the 28-day PC-PEP intervention’s effectiveness at improving physiological relaxation on cue as measured by brain wave activity (Muse™) and HRV indices (HeartMath^®^) among a group of PCa survivors from the Maritimes, Canada. During a 28-day trial period, participants engaged in a multifaceted health promotion program that included various activities aimed at stress reduction, physical fitness, pelvic floor muscle training, intimacy, and connection, as well as peer support and accountability.

### 4.1. Brain Wave Activity

Interactions between Time (pre vs. post) and Sensor Scalp Assessment Times (every 30 s for 7 min of testing) emerged as statistically significant and overall showed that delta, theta, alpha, beta, and gamma brain wave amplitudes were greater in the first half of the post-intervention testing time (first 3 to 4 min) when compared to baseline assessments, but similar with baseline assessments for the second half (last 4 to 7 min). According to Dudeja and Scientific American, delta waves are typically more prominent during sleep or deep relaxing meditation, indicating lower arousal and decreased consciousness [39,44]. Theta waves, associated with increased creativity, relaxation, emotional connection, and daydreaming, have been shown to increase during stress-reduction activities like yoga and tai chi, as mentioned by Dudeja and The Science of Brainwaves [40,44]. Alpha wave activity has been observed to increase during periods of rest and relaxation, including meditation, as discussed in studies by Braboszcz et al. and Dziembowska et al. [45,46]. Increased beta wave activity indicates high arousal and the formation of complex thoughts [39,40]. Finally, gamma wave activity during meditation was found to be associated with diverse cognitive functioning and heightened perception [45]. As such, increases in each of these wave amplitudes has the potential to indicate effective meditation. However, it is important to note that the length and depth of meditative states can vary among individuals, and the progression may also depend on various factors such as meditation technique, frequency of practice, and individual differences in mental and physiological characteristics [47,48,49].

Research suggests that increased periods of meditation practice can lead to longer durations of meditative states. Regular and consistent meditation practice over time can enhance an individual’s ability to enter deeper states of meditation and sustain them for longer periods [49]. As practitioners become more experienced, they often develop greater focus, concentration, and mindfulness, which contribute to prolonged meditative states [48]. This ability has typically been linked with frontal lobe activation [48]. Indeed, after the PC-PEP intervention was completed, on average, our results indicated that alpha wave activity was higher in the two frontal scalp sensor locations, but not in the temporal scalp locations. A recent meta-analysis demonstrated that meditation has an impact on the prefrontal cortex, which could explain the increased alpha wave activity from the two frontal scalp sensors (but not the two temporal sensors) at post-intervention, compared to pre-intervention measurements revealed by the three-way Time × Sensor Scalp Assessment Time and Sensor Scalp Location interactions that were observed [50]. 

While all brain waves had statistically significant increases in amplitudes post-intervention compared to pre-intervention, it is noteworthy that these increases were observed primarily during the first half of the 7-min meditation period. In other words, the post-intervention measurements showed higher amplitudes for all brain waves, but this effect was more pronounced in the initial half of the meditation session. This observation may be attributed to the relatively short duration (28 days) of the PC-PEP intervention, which may not have allowed participants enough time to achieve significant improvements in their meditation skills throughout the entire meditation exercise. We hypothesize that a longer intervention period would likely yield even greater results that could be sustained for the entire duration of the meditation practice. This hypothesis is currently being tested in subsequent 6-month iterations of the PC-PEP program.

Furthermore, it is important to note that the compliance rate for the meditation component of PC-PEP differed in terms of the frequency of daily engagement. When considering the overall number of minutes of weekly meditation, their compliance was very high, with an average of 66.23 min per week out of the recommended 70 min (95% compliance rate) [29]. However, on average, participants completed 5 days of meditation per week out of the prescribed 7 days, resulting in a compliance rate of 71% [29]. Considering these daily compliance rates, it is plausible that participants may not have fully maximized the potential benefits of the meditation component, which could contribute to the transient nature of the observed changes in brain wave activity.

### 4.2. Muse™ Metrics 

Based on the analysis of participants’ brain waves captured by Muse™ EEG outputs, it was observed that post-PC-PEP intervention, they showed a slightly more efficient ability to reach and sustain calm states. Additionally, they exhibited marginal improvements in their ability to refocus their attention on their breathing after becoming distracted during meditation [41]. However, it is important to note that these results did not reach statistical significance and would benefit from reassessment in future studies with longer intervention durations, providing more time for progressive improvements in meditation technique.

### 4.3. HeartMath^®^ Heart Rate Variability Metrics

With marginal significance, participants exhibited higher HeartMath^®^ metrics (achievement score), which are associated with greater HRV, after the PC-PEP intervention. An achievement score of 300 or above is considered a good baseline target when starting a meditation intervention and, on average, participants surpassed this threshold in their follow-up assessments [42]. Higher HRV is associated with positive emotions such as love and appreciation, while lower HRV is linked to stress, anger, and frustration [42]. Numerous studies have demonstrated increased HRV following meditation interventions [51,52,53]. However, when considering other HRV metrics provided by HeartMath^®^, including Low Coherence %, Medium Coherence %, High Coherence %, and Average Coherence, there was a lack of statistical significance. Similar to the findings in brain wave metrics, we believe that the absence of statistical significance in HRV metrics could be attributed to the relatively short duration of the intervention. Participants were required to familiarize themselves with the Inner Balance™ infrared pulse sensor attached to their earlobe for this exercise. It is possible that challenges adjusting to this new equipment may have also impacted the efficacy of the exercise and, therefore, the results. Despite this, all metrics showed positive trends, suggesting that with more time to practice meditation and become accustomed to the equipment, participants may demonstrate significant improvements in HRV, as observed in similar studies [51,52,53].

### 4.4. Limitations

This phase 2 trial has certain limitations that should be acknowledged. Firstly, the sample size was relatively small, consisting of 30 PCa survivors, and may not be representative of the wider population. Participants in the study were predominantly white, highly educated, had higher socioeconomic backgrounds, and were married. Therefore, the findings may not generalize to more diverse populations.

Secondly, due to the nature of being a feasibility trial, the intervention duration was limited to 28 days. Subsequent phases of the trial have addressed this limitation by examining the effects of a 6-month version of the PC-PEP intervention over a period of 12 months to gain a better understanding of its benefits [30]. Furthermore, ongoing analyses are being conducted to assess the impact of the stress reduction component of the program long term [30]. It should also be noted, however, that a longer intervention (i.e., 6 months) imposes a greater time commitment and potential burden for participants [30].

It is important to note that this paper focused exclusively on the meditation aspect of PC-PEP, using Muse™ EEG outputs and HeartMath^®^ HRV metrics to measure stress reduction. However, it is worth acknowledging that other components of the program, including exercise, pelvic floor strengthening, intimacy and connection, and peer support and accountability, have the potential to reduce stress as well. For instance, improvements in symptoms of urinary incontinence and erectile dysfunction resulting from the progressive pelvic floor exercise program may significantly alleviate major stressors experienced by participants prior to the intervention. Future papers will aim to explore the contributions of these various components of the 28-day and longer versions of the PC-PEP program to enhance participants’ overall quality of life. The findings of this study have the potential to guide enhancements in future versions of the PC-PEP intervention, aiming to optimize the benefits experienced by patients with PCa.

## 5. Conclusions

The implications of this study’s findings hold significant relevance for the well-being of prostate cancer survivors. The investigation aimed to determine the efficacy of a 28-day iteration of the PC-PEP program in eliciting a controlled reduction of stress response, comparing the conclusion to the initiation of the intervention. Although statistical significance was not consistently reached within the examined dataset, the results consistently manifested a favorable trajectory towards diminished stress levels after the intervention, particularly during the initial phase of testing rather than the latter. In the initial testing phase, discernible enhancements in brain wave activity profiles indicated an augmented capacity to attain and sustain states of tranquility. Likewise, metrics related to heart rate variability exhibited encouraging patterns synonymous with stress reduction during this period. Notwithstanding the study’s limitations associated with the small cohort size and relatively brief intervention duration, the constructive outcomes underscore the latent benefits of PC-PEP for alleviating stress among prostate cancer survivors. The study’s outcomes lay the groundwork for protracted interventions to optimize participant outcomes and provide direction for subsequent program refinements. In addition, given that the stress-reducing HRV biofeedback device was not allocated for home use during the intervention, the present findings suggest the potential necessity of the equipment itself for consistent and statistically significant improvements in pre-to-post intervention stress levels. This proposition will be reevaluated in Phase 3 and 4 trials, encompassing an extended 6-month version of the intervention. Specifically, forthcoming trials provide participating patients with a biofeedback HRV monitor throughout the 6-month duration of the PC-PEP trials. If the inclusion of the biofeedback device indeed culminates in substantial pre-to-post-intervention stress reduction relative to the standard of care, then the financial implications of integrating an HRV device should be considered when evaluating the implementation costs of the intervention in the context of medical standards of care. Conversely, if the device does not yield significant stress reduction disparities, the associated costs will be notably curtailed (the device costs approximately CAD 200). Regardless, considering the noteworthy outcomes previously observed in diminishing psychological distress and augmenting physical function within the broader context, this study underscores the potential of holistic modalities like PC-PEP in heightening cancer survivors’ quality of life and well-being [29,30]. This suggests that the PC-PEP intervention is valuable in achieving its intended objectives and lays the groundwork for extending the program’s duration to enhance participant outcomes to a greater extent.

Finally, pre-treatment stress reduction interventions might offer potential support for post-treatment pelvic floor recovery. This is attributed to patients acquiring relaxation techniques for all body muscles, encompassing pelvic floor muscles. These acquired skills could contribute to their recuperation from post-treatment urinary leakage, especially during instances of strain, where pelvic floor muscle training through kegel exercises is typically advocated within urology departments as an integral facet of standard care. 

Such a psychological preparation would fit in the multimodal pre-habilitation process advocated by Silver and Baima (2013) [54]. 

## Figures and Tables

**Table 1:** Three-way (Time × Sensor Scalp Location × Sensor Scalp Assessment Time) repeated-measures ANOVA assessing delta, theta, alpha, beta, and gamma waves brain waves using Muse™ biofeedback among a sample of 30 PCa survivors from the Maritimes, Canada.

Delta Waves	Test Statistic ^1^	*p* Value	η^2^	Power
Time	F (1, 27) = 0.99	0.33	0.04	0.16
Sensor Scalp Location	F (2.11, 57.05) = 19.28	<0.001	0.42	1
Sensor Scalp Assessment Time	F (4.38, 118.22) = 11.20	<0.001	0.29	1
Time × Sensor Scalp Location	F (2.15, 57.95) = 0.37	0.71	0.01	0.11
Time × Sensor Scalp Assessment Time	F (4.67, 126.03) = 4.06	0.002	0.13	0.94
Time × Sensor Scalp Location × Sensor Scalp Assessment Time	F (8.55, 230.84) = 1.25	0.27	0.04	0.59
Sensor Scalp Location × Sensor Scalp Assessment Time	F (9.90, 267.20) = 0.56	0.84	0.02	0.29
**Theta Waves**	**Test Statistic**	***p* Value**	**η^2^**	**Power**
Time	F (1, 27) = 1.94	0.18	0.07	0.27
Sensor Scalp Location	F (1.97, 53.18) = 81.79	<0.001	0.75	1
Sensor Scalp Assessment Time	F (4.27, 115.15) = 6.74	<0.001	0.20	0.99
Time × Sensor Scalp Location	F (2.03, 54.80) = 0.57	0.57	0.02	0.14
Time × Sensor Scalp Assessment Time	F (4.67, 126.15) = 4.14	0.002	0.13	0.94
Time × Sensor Scalp Location × Sensor Scalp Assessment Time	F (7.35, 198.44) = 1.03	0.42	0.04	0.45
Sensor Scalp Location × Sensor Scalp Assessment Time	F (8.86, 239.08) = 1.42	0.18	0.05	0.67
**Alpha Waves**	**Test Statistic**	***p* Value**	**η^2^**	**Power**
Time	F (1, 27) = 1.68	0.21	0.06	0.24
Sensor Scalp Location	F (2.07, 55.87) = 225.15	<0.001	0.89	1
Sensor Scalp Assessment Time	F (3.41, 92.17) = 0.90	0.45	0.03	0.26
Time × Sensor Scalp Location	F (2.04, 55.11) = 0.62	0.55	0.02	0.15
Time × Sensor Scalp Assessment Time	F (4.69, 126.60) = 2.44	0.04	0.08	0.74
Time × Sensor Scalp Location × Sensor Scalp Assessment Time	F (7.62, 205.73) = 2.09	0.04	0.07	0.82
Sensor Scalp Location × Sensor Scalp Assessment Time	F (7.65, 206.52) = 2.60	0.01	0.09	0.91
**Beta Waves**	**Test Statistic**	***p* Value**	**η^2^**	**Power**
Time	F (1, 27) = 0.65	0.43	0.02	0.12
Sensor Scalp Location	F (1.86, 50.11) = 39.72	<0.001	0.60	1
Sensor Scalp Assessment Time	F (2.81, 75.97) = 0.85	0.47	0.03	0.22
Time × Sensor Scalp Location	F (1.94, 52.40) = 0.64	0.53	0.02	0.15
Time × Sensor Scalp Assessment Time	F (4.83, 130.32) = 4.48	<0.001	0.14	0.96
Time × Sensor Scalp Location × Sensor Scalp Assessment Time	F (7.22, 194.92) = 1.77	0.09	0.06	0.72
Sensor Scalp Location × Sensor Scalp Assessment Time	F (5.20, 140.30) = 1.33	0.25	0.05	0.47
**Gamma Waves**	**Test Statistic**	***p* Value**	**η^2^**	**Power**
Time	F (1, 27) = 1.82	0.19	0.06	0.26
Sensor Scalp Location	F (1.95, 52.65) = 4.49	0.02	0.14	0.74
Sensor Scalp Assessment Time	F (2.88, 77.72) = 2.50	0.07	0.09	0.59
Time × Sensor Scalp Location	F (1.70, 46.02) = 0.80	0.44	0.03	0.17
Time × Sensor Scalp Assessment Time	F (3.43, 92.72) = 3.62	0.01	0.12	0.82
Time × Sensor Scalp Location × Sensor Scalp Assessment Time	F (6.82, 184.26) = 0.99	0.44	0.04	0.41
Sensor Scalp Location × Sensor Scalp Assessment Time	F (5.30, 143) = 2.37	0.04	0.08	0.76

^1^ Using the Greenhouse–Geisser adjustment to the degrees of freedom.

**Table 2:** Repeated-measures ANOVA assessing the impact of the PC-PEP intervention on the ability of 30 male PCa survivors to meet meditation milestones as measured from Muse™ metrics from the Maritimes, Canada.

Muse™ EEG Outputs	Estimated Marginal Means		Test Statistic	*p* Value	η^2^	Power
	Pre-Intervention	Post-Intervention				
Calm	1048.80	1062.40	F (1, 29) = 0.03	0.87	0.001	0.05
Neutral	236.83	212.33	F (1, 29) = 1.47	0.24	0.05	0.22
Recoveries	8.13	10.33	F (1, 29) = 0.81	0.38	0.03	0.14
Birds	41.73	46.53	F (1, 29) = 0.86	0.36	0.03	0.15

**Table 3:** Repeated-measures ANOVA of heart rate variability metrics in a sample of 30 male PCa survivors from the Maritimes, Canada.

Heart Rate Variability	Estimated Marginal Means		Test Statistic	*p* Value	η^2^	Power
	Pre-Intervention	Post-Intervention				
Low Coherence %	6.23	5.27	F (1, 29) = 0.89	0.35	0.03	0.15
Med Coherence %	11.23	11.10	F (1, 29) = 0.01	0.94	0.00	0.05
High Coherence %	82.53	83.63	F (1, 29) = 0.22	0.64	0.01	0.07
Average Coherence	2.68	2.93	F (1, 29) = 1.60	0.22	0.05	0.23
Achievement Score	315.77	372.20	F (1, 29) = 3.49	0.07	0.11	0.44

## Data Availability

Data from this study are available to researchers through a data access process in compliance with the Patient Privacy and Protection Research Act (NSHA Research Ethics Board).

## Supplementary Materials

The following supporting information can be downloaded at: https://www.mdpi.com/article/10.3390/curroncol30090577/s1, Table S1: Demographic Characteristics of PC-PEP Feasibility Study Sample.
