# Supplementary material for: Changes in Stress Reduction Following a 28-Day Prostate Cancer Patient Empowerment Program (PC-PEP) among Prostate Cancer Survivors
Source: Curr Oncol. 2023 Aug 29;30(9):7936–49. doi: 10.3390/curroncol30090577 (PMC10528192; doi:10.3390/curroncol30090577)
Supplement: Supplementary file 1 [file curroncol-30-00577-s001.zip › curroncol-2547348-supplementary.pdf]

Table S1. Demographic characteristics of the PC-PEP feasibility study among 30 men with a history of prostate cancer from the Maritimes, Canada.

Sex	Male: n=30 (100%), Female n=0 (0%)
Age	Mean: 68.93 years, Range: 56-83 years old
Ethnicity	White/Caucasian: n=28 (93%)
Education	University: n=20 (67%)
Relationship status	In a relationship: n=30 (100%)
Employment status	Retired or unemployed: n=21 (70%)
Household income	Less than 80K/year: n=8 (27%)
Time between diagnosis and survey	More than 25 months: n=22 (73%)
Type of treatment for PCa	Active surveillance: n=4 (13%)<br>Radical prostatectomy: n=14 (47%)<br>Radiation (beam, brachy or seed) +/- hormone: n=6 (20%)<br>Radical prostatectomy, radiation and hormones: n=4 (13%)<br>Androgen Depravation Therapy: n=2 (7%)
